# Study on Dynamic Recrystallization under Thermal Cycles in the Process of Direct Energy Deposition for 316 L Austenitic Stainless Steel

**DOI:** 10.3390/ma17194860

**Published:** 2024-10-02

**Authors:** Manping Cheng, Xi Zou, Tengfei Chang, Lehui Liu

**Affiliations:** 1 School of Intelligent Manufacturing and Mechanical Engineering, Hunan Institute of Technology, Hengyang 421002, China; 2 School of Mechanical and Electrical Engineering, Hunan University of Science and Technology, Xiangtan 411201, China

**Keywords:** additive manufacturing, dynamic recrystallization, thermal cycles, 316 L stainless steel

## Abstract

In the process of directed energy deposition (DED), the grain structure of the deposited samples is determined by two aspects. The first is the initial solidification grain structure; the second is the effect of the upper thermal cycle on the solidified grain structure of the lower layer. Dynamic recrystallization and grain growth can be activated under suitable strain and the temperature resulting from thermal cycles. The evolution of grain size and the geometric dislocation density (GND) of austenitic stainless steel 316 L under different strains and temperatures caused by thermal cycles was investigated. It is found that dynamic recrystallization requires an appropriate level of accumulated strain, temperature, and initial grain size. Under <2% accumulated strain and 400–1200 °C conditions caused by 30 layers of thermal cycles, fully dynamic recrystallization occurs with coarse initial grains (CIG), leading to the complete coarsening of grains. However, relatively fine initial grains (FIG) under the same conditions only display partial dynamic recrystallization. The next 2–4% strain and 400–700 °C by 60 layers of thermal cycles make up the driving force of fully dynamic recrystallization, and the grains coarsen completely. Larger accumulated strain (4–6%) and lower temperature (400–600 °C) by 90 layers of thermal cycles and FIG provide more nucleation sites for dynamic recrystallization, which leads to little coarsening of grains even after fully dynamic recrystallization. Temperature, accumulated strain, and the amount of δ-ferrite promote the formation of sub-grains during dynamic recrystallization caused by thermal cycles, which leads to the increase in GND.

## 1. Introduction

Laser additive manufacturing has the advantages of high flexibility, a short processing cycle, the ability to form complex shapes for structural parts, and so on [1]. In this process, powder particles are melted onto deposited samples. It is produced via the method of “layered printing and layer by layer stacking” through software and a numerical control system [2]. During DED, the rapid heating and cooling of the upper laser will inevitably bring thermal strain to the lower solidified material due to the existence of thermal cycle [3,4,5]. The continuously accumulated thermal strain and temperature can provide favorable conditions for the occurrence of dynamic recrystallization (DRX) and dynamic recovery (DRV) [6,7]. These phenomena can be explained and described by the Zener–Hollomon equation [8]. The traditional dynamic recrystallization depends on the strain, strain rate, and temperature. The large strain leads to the increase in the recrystallization fraction and fine grains [9,10], but the small strain is not enough to provide the energy required for DRX nucleation, and DRX is difficult to accomplish. Both a high strain rate and a low strain rate are favorable for DRX [11]. Fengming Qin [12] studied the recrystallization mechanism under an extreme strain rate. At a low strain rate (below 10^−1^s^−1^), the recrystallization mechanism is dislocation slip and climbing, for it has a longer time to promote dislocation movement, which leads to DRX. At a high strain rate (10^−1^s^−1^), the recrystallization mechanism is the Luan crystal mechanism; a deformed Luan crystal is generated, providing a recrystallization nucleation point. Meanwhile, temperature has a greater effect on the recrystallized grain size relative to strain [13].

Different from conventional DRX, both thermal strain and temperature are not constant due to the transient and varying nature of thermal cycles during additive manufacturing (AM), while the strain rate is kept at the low strain rate (10^−2^s^−1^) from the perspective of traditional dynamic recrystallization [6]. Hu [14] found substantial recrystallized grains in the middle region of the deposited sample, while abundant sub-grains resulting from the rearrangement of dislocation were discovered at the bottom. Zhang [15] also discovered that through thermal cycles, a large number of dislocations caused by strains were consumed at the bottom of the deposited sample to refine the grains, and this phenomenon is due to DRX. Sabzi [16] found fine grains caused by thermal cycles at the bottom of the deposited sample, and this provided sufficient evidence and a mechanism explanation for dynamic recrystallization. Its behavior was also affected by laser process parameters. The large linear energy density leads to the occurrence of continuous dynamic recrystallization (CDRX), and high-angle grain boundaries (HAGBs) develop in the microstructure. However, most grains undergo incomplete continuous dynamic recrystallization (DDRX) and dynamic recovery (DRV), developing low-angle grain boundaries (LAGBs). This is suggested as the main approach for yield strength improvement [17]. Huang [18] further pointed out the thermal conditions required by different recrystallization mechanisms during AM.

However, to our knowledge, in the process of AM, the bottom region of the deposited sample often shows different undercooling compared with other regions, which cause diverse initial grain sizes [19,20]. In the traditional dynamic recrystallization theory, recrystallized grains and dislocation density show diverse morphologies under distinct conditions including initial grain size, strains, and temperature [21]. Although these articles mentioned above displayed various grain microstructures after dynamic recrystallization caused by thermal cycle, and explained the mechanism to a certain extent, they did not take into account the effect of initial grain size on recrystallized grain morphology and dislocation density at the quantified thermal strain and temperatures caused by thermal cycles. These three key indicators (initial grain size, thermal strain, and temperature) are also still lacking information. The aim of the present study is to explore the effects of initial grain size after solidification, in situ strain, and temperature caused by thermal cycles on the recrystallized grain size and dislocation density, and its underlying mechanisms, so as to expect the possibility of achieving in situ grain refinement and a high-dislocation density microstructure.

## 2. Materials and Methods

This experiment used commercially available 316 L stainless steel provided by the Changsha Tianjiu Metal Materials Company. Its particle size is 45–125 μm. The chemical composition is shown in Table 1. Powder was processed in a vacuum drying chamber before the experiments. The chamber’s temperature had to be adjusted to 120 degree centigrade within 20 min in order to ensure that the moisture in powder was completely removed. The samples were directly fabricated using a YLS-4000-CL laser direct deposition system with a continuous 1070 nm wavelength fiber laser. The laser equipment is provided by Dazu Laser Technology Co., Ltd. (Shenzhen, China). The laser power was set to 800 W, the beam diameter to 1.2 mm, the hatch spacing to 0.75 mm, the powder layer thickness to 0.2 mm, the scanning speed to 8 mm/s, and the powder feeding rate to 11.5 g/rad. All samples were printed in an argon atmosphere under 0.3 MPa pressure.

**Figure 1:**
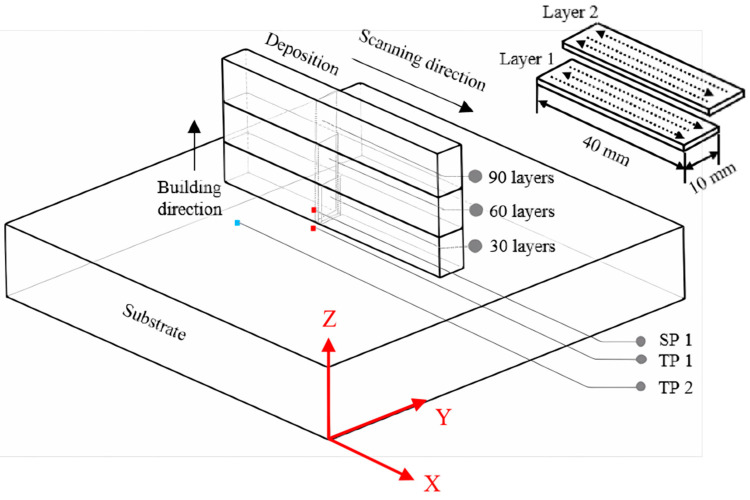
Sample size and scanning path.

Horizontal back and forth linear scanning paths were utilized, with a 180° scan vector rotation on the next layer, which finally formed 40 mm long, 6 mm wide samples. The height of the sample is determined by the number of printing layers, and three samples of different heights were manufactured (5.5 mm/11 mm/17 mm for 30/60/90 layers, respectively), as shown schematically in Figure 1. The relative density of the as-deposited samples in our work was above 99.8%.

In order to monitor the temperature variation in the deposition process in situ, two thermocouples were placed on the substrate. The point of the first thermocouple is located at the midpoint of the first deposition layer in the longitudinal direction (X direction), with a vertical distance of about 0.5 mm in the transverse direction (Y direction), called temperature point 1 (TP1). The point of the second thermocouple is located at one-third of the first deposition layer in the longitudinal direction, with a vertical distance of about 2 mm in the transverse direction, called temperature point 2 (TP2), as shown by the red dots in Figure 1. An industrial camera (Balser company, Monheim am Rhein, Germany) was used to obtain in situ strain data using the digital image correlation (DIC) method [22,23], and X-strain data at a position 1 mm above the deposition/substrate interface were extracted and called SP 1, as shown by the blue dots in Figure 1. The specimens along the cross-section perpendicular to laser scan direction were cut. The grain size, grain boundary, and geometric dislocation density (GND) of nine regions in three as-deposited samples with different locations from the substrate (bottom/middle/top regions of 30/60/90 layers) were observed using EBSD. The cross-sections of the samples were firstly polished with 800#-1200#-2000# grade sandpaper and then polished with 1 μm diamond paste. Finally, 8% perchloric acid and 92% ethanol mixed electrolyte were used to electropolish at 20 V DC for 40 s. The step size is 3 μm in the range of 750 μm × 750 μm. The EBSD data were analyzed by Channel 5 (by Oxford Instruments) and MTEX open-source software, which can be installed in the MATLAB toolbox. These data were used to examine the effect of dynamic recrystallization, which is caused by subsequent thermal cycles, on grain size and GND under different temperature and strain conditions. According to the grain misorientation, low-angle grain boundaries (LAGBs) and high-angle grain boundaries (HAGBs) are determined as follows: angles of 2° to 15° are defined as LAGBs, while HAGBs displays misorientation angles > 15° [14]. The metallographic picture with δ-ferrite and γ-austenite phases of the same regions as mentioned above were characterized on etched cross-sections by means of scanning electron microscopy (SEM), in conjunction with energy-dispersive spectroscopy (EDS). Aqua regia, which is a mixture of concentrated hydrochloric acid (HCl) and concentrated nitric acid (HNO ₃) in a volume ratio of 3:1, was used to etch the cross-section surface polished with sandpaper and diamond paste for about 9 s. The solidification mode of the crystal can be preliminarily determined by observing the composition of δ-ferrite, γ-austenite, and the ratio of Cr eq. and Ni eq. in SEM data. The EBSD-SEM-EDS testing equipment was a Helios Nanolab 600i.

## 3. Results

### 3.1. In Situ Temperature and Strain Measurement

**Figure 2:**
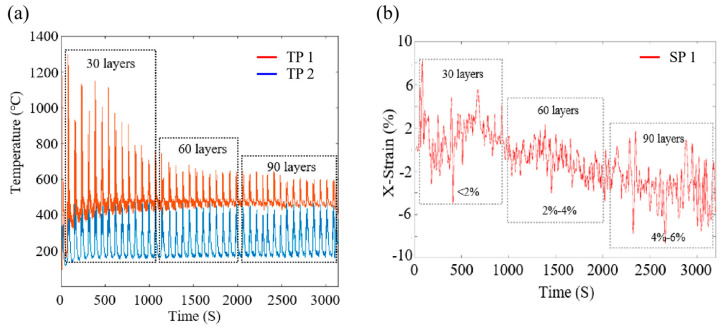
(**a**) Thermal history of 90 layers of deposition at TP 1 and TP 2; (**b**) accumulated strain of 90 layers of deposition at SP 1.

The heat is quickly transferred from the molten pool to other metal positions due to heat conduction, which leads to regular fluctuations in the temperature of the monitoring points (TP 1 and TP 2). Figure 2a shows the evolution of temperature in TP 1 and TP 2. In the first 30 layers, the maximum temperature can reach about 1200 °C. In the vertical direction, the higher the number of layers printed, the lower the temperature of TP 1 due to the increase in the distance from the heat source. The maximum temperature drops to approximately 650 °C during the last 60 layers of deposition. It is worth noting that the TP1 temperature is always retained above 400 °C in the 90-layer deposition. Because TP 2 is far away from the heat source in the transverse direction, its temperature is always balanced between 200 °C and 400 °C. In the following discussions, the temperature history data of TP 1 could be used to analyze the effect of the thermal cycles on the microstructural evolution of grains obtained by EBSD and SEM. Figure 2b displays the X-strain of SP 1. The maximum accumulated strain at SP 1 is about 2% in the first 30 layers, and in the next 60 and 90 layers of deposition, the cumulative strain is approximately 2–4% and 4–6%, respectively. The accumulated increased strain is due to the plastic deformation of the material, which is caused by the thermal expansion and contraction. In the following discussions, accumulated strain will be used as an important parameter to influence the microscopic evolution of grains.

### 3.2. As-Deposited Grain Size

In the process of deposition, there are two factors that affect the final microstructure. First of all are the solidification conditions. In a standard fast-cooled austenitic stainless steel 316 L, austenite (γ) and δ-ferrite precipitate depending on the steel’s chemical composition [24,25]. The content of precipitated austenite and ferrite determines the nucleation mode by calculation of the element Cr/Ni ratio, which leads to various microstructural features [26]. The cooling rate R during precipitation determines the primary grain morphology and size [27,28]. The second factor that affects the final microstructure is dynamic recovery and dynamic recrystallization. The printing of the upper layers brings cyclic reheating to the lower layers, and the accumulated thermal strain and temperature caused by thermal cycles probably affects the initial solidified microstructural [29,30].

**Figure 3:**
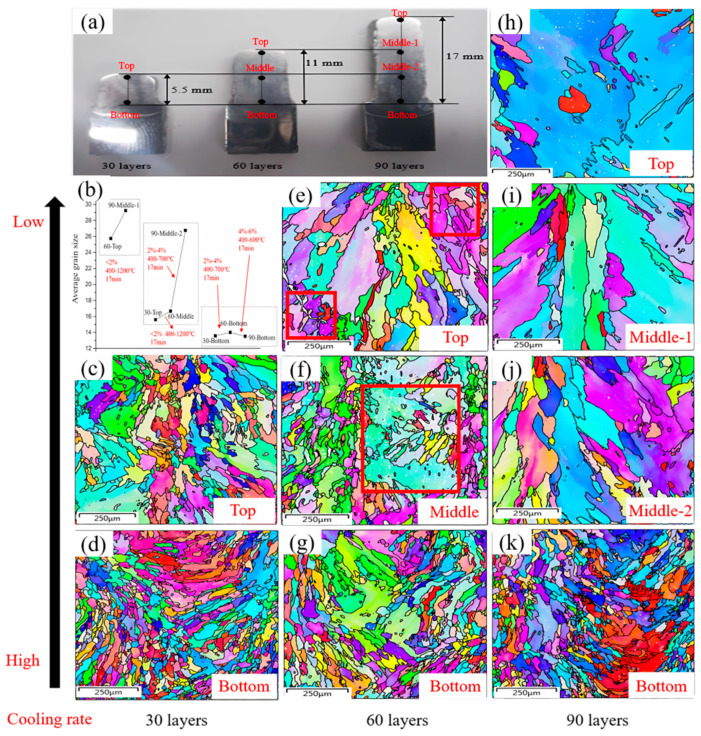
(**a**) Location of the nine measuring regions of three samples. (**b**) The evolution of the average grain size under thermal cycles with the same cooling rate. (**c**–**k**) EBSD grain boundary map of 10 measuring regions of 3 as-built samples.

In order to explore the effect of accumulated strain and temperature caused by thermal cycles, the nine regions are divided, as shown in Figure 3a. Three groups are compared. The first group has 60 layers at the top and 90 layers in the middle, which experience 30 thermal cycles for about 17 min; the second group has 30 layers at the top, 60 layers in the middle-1 section, and 90 layers in the middle-2 section, which undergo 60 thermal cycles for about 34 min; the third group is has 30 layers at the bottom, 60 layers at the bottom, and 90 layers at the bottom, which go through 90 thermal cycles for about 51 min. It should be noted that the cooling rate of the third group is greater than those of the first and the second groups due to it being closer to the substrate [10]. However, the group’s internal cooling rates are the same. Therefore, the initial grain size of the third group is much smaller than that of the other two groups.

The change in average grain size in the three groups is shown in Figure 3b. After thermal cycles, the grain size improved to various degrees. Its variability depends on the accumulated strain and temperature caused by the thermal cycles, which leads to dynamic recrystallization and recovery. The grain size and grain boundary of nine regions are shown in Figure 3c–k, which indicates that the as-deposited microstructure display columnar dendrites accompanied by equiaxed crystals with a nearly random misorientation. This is typical in additive manufacturing [31,32]. As the bottom layer near the substrate has a greater cooling rate than other regions, this brings about a higher undercooling and nucleation rate [10]. Thus, the grain sizes in the bottom regions in the 30/60/90 layers are smaller than those in the middle and top regions, respectively. It is worth mentioning that from the image, the grain size of the top region of 30 layers is much smaller than that of the top regions of 60 and 90 layers, which may be due to the fact that the 60-layer and 90-layer sections have experienced more respective thermal insulation processes of the bottom layer. This is equivalent to the preheating effects [33], and reduces the cooling rate and increase the grain size.

The effects of the accumulated strain and temperature fluctuation on grain size under the same cooling rate were considered. As for the first group, with 60 layers at the top and 90 layers in the middle-1 section, which experience 30 thermal cycles for 17 min, the accumulated strain and temperature in Figure 2 are <2% and 400–1200 °C, respectively. Under this condition, some small grains disappear and merge with large grains with a long columnar shape, as shown in Figure 3e (red box) and Figure 3i. The average grain size increases from 26 μm to approximately 30 μm. For the second group, with 30 layers at the top, 60 layers in the middle, and 90 layers in the middle-2 section (60 thermal cycles for about 34 min), the grain grows partially in a relatively small strain and large temperature fluctuation condition (<2%/400–1200 °C), as shown in Figure 3e,f (red box). Then, the accumulated strain increases to 2–4%, and the temperature range decreases to 400–650 °C, which results in complete grain coarsening and the formation of coarse columnar grains, as shown in Figure 3j. The average grain size increases from 15 μm to 17 μm, and then increases to 27 μm. For the third group, the bottom regions of the 90-layer samples go through a relatively large accumulated strain (4–6%) and a low temperature fluctuation (400–600 °C) for about 51 min. As seen in Figure 3d,g,k, the grains change only slightly, and the average grain size is relatively stable at 13 μm in the whole 90-layer deposition.

### 3.3. As-Deposited GND

**Figure 4:**
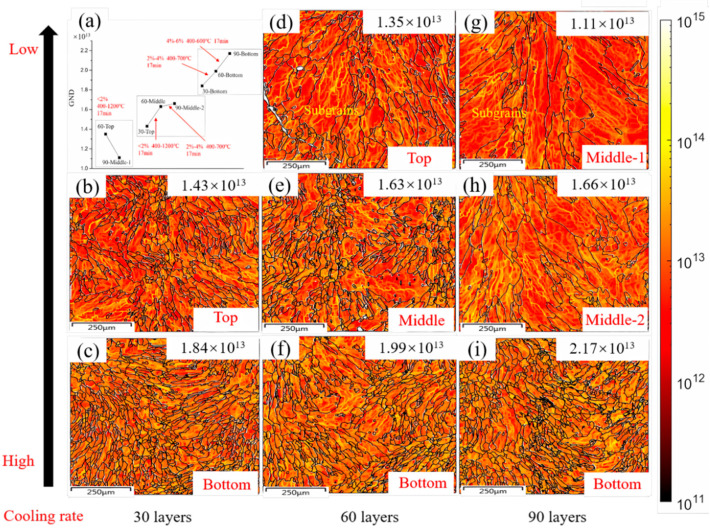
(**a**) The evolution of GND under thermal cycles with the same cooling rate; (**b**–**i**) the GND of three groups of as-built samples.

Figure 4b–g shows a comparison of GND for eight regions in the three groups, and the GND values are extracted for comparative analysis, as shown in Figure 4a. For the first group, with 60 layers at the top and 90 layers in the middle-1 section, the GND decreases slightly, from 1.35 × 10^13^ down to 1.11 × 10^13^, as shown in Figure 4d,g, which is due to grain coarsening caused by dynamic recrystallization, as mentioned above. Generally, the movement of dislocations cannot pass through the grain boundary [34,35]. The courser the grain, the smaller the grain boundary, and the worse its ability to capture dislocations [36]. On the other hand, 400–1200 °C high-temperature fluctuations would increase the movement ability of dislocations. Once the dislocations moving on different slip systems meet under certain strain, they undergo delivery and pinning, and the continuous pinning of dislocations causes dislocation stacking and gradually forms sub-grains [37], which are also an important source of GND [38]. As shown in Figure 4, the yellow lines interspersed in the grains increase the GND. However, in the process with 60 layers at the top and 90 layers in the middle-1 section, with 17 min thermal cycles, the accumulation of dislocations caused by sub-grains cannot completely supplement the annihilation of dislocations caused by the decrease in the grain boundaries. Thus, the GND of the 60 layers at the top is smaller than that of the 90 layers in the middle-1 section. For the second group, with 30 layers top at the top and 60 layers in the middle, the GND exhibits a rising trend to a small extent (1.43 × 10^13^ and 1.63 × 10^13^, respectively), which is opposite to that of group 1, as shown in Figure 4b,e. It is worth mentioning that although it is under the same conditions with 2% accumulated strain and a 400–1200 °C temperature fluctuation as group 1, the grain size changes only slightly. Therefore, the increase in GND is entirely contributed by the formation of sub-grains, and the GND formed at the grain boundary is basically unchanged. Then, for the 60 layers in the middle and 90 layers in the middle-2 section, an accumulated 2–4% strain is a key factor to bring about sub-grains, results in an improvement in GND and neutralizing the decrease in GND caused by grain coarsening. As a result, the GND remains stable, at about 1.65 × 10^13^, as shown in Figure 4e,h. For the third group, at a stable temperature fluctuation of about 400–600 °C and an increasing accumulated strain from 2% to 4–6%, the GND growth steadily increases from 1.84 × 10^13^ to 2.17 × 10^13^. This is due to the multi-point nucleation caused by the accumulated large strain and the grain refinement after dynamic recrystallization. The 90 layers at the top no longer undergo any thermal cycles in this experiment. In this section, the effect of the three groups with different numbers of thermal cycles on GND is discussed. Therefore, the 90-layer top is not considered.

### 3.4. As-Deposited Microstructures

**Figure 5:**
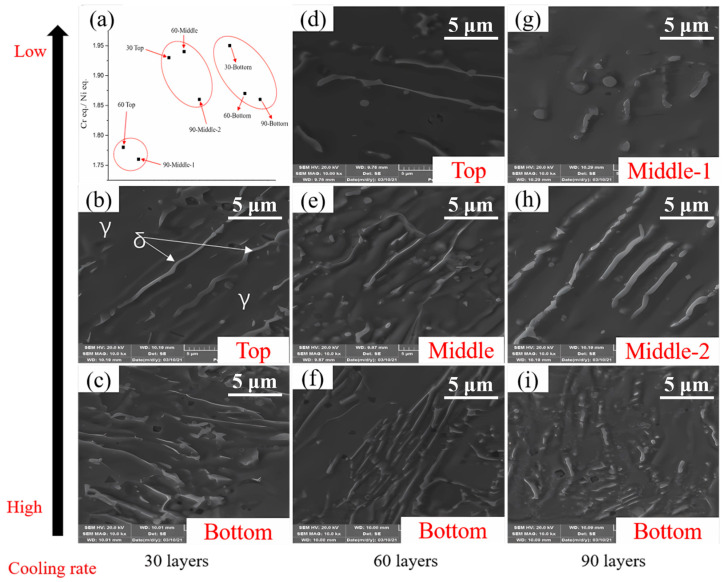
(**a**) The equivalents of Cr and Ni and their ratio as determined by SEM. (**b**–**i**) SEM images of the evolution of microstructures of the three groups of as-built samples.

In standard austenitic steel with rapid cooling, according to the chemical composition during solidification, two kinds of microstructure constituents can be obtained: δ-ferrite and γ-austenite. The as-deposited microstructures of three groups are revealed by SEM in Figure 5b,i. The white strip is δ-ferrite, as indicated by the white arrow, and the rest is γ-austenite [25]. For the comparison of the three groups, the first group displays less δ-ferrite, while the second and third groups exhibit more, which indicates that the δ-ferrite content increases and the γ-austenite decreases from the substrate to the top of the deposited. The quantities of δ-ferrite and γ-austenite are determined by the solidification mode, decided by the ratio of equivalent of Cr and Ni in rapid-cooled austenitic stainless steel from the solidification temperature to room temperature according to the cooling rate [26]. This was obtained and calculated by scanning with EDS and SEM, as shown in Figure 5a. The ratio of Cr eq. and Ni eq. in group 2 and group 3 (about 1.9) is much more than that in groups 1 (approximately 1.76), resulting in more δ-ferrite and less γ-austenite. Primary δ-ferrite precipitates in the fusion zone, with γ stabilizing elements enriched in the liquid which then solidifies into the γ structure. However, Lath δ-ferrite plays an important role in pinning motive dislocation under appropriate temperature fluctuation and strain conditions [37], resulting in an increase in GND, which can also explain why the GND of the second group increased and that of the first group reduced. This will be detailed in the discussion section.

## 4. Discussion

### 4.1. Conditions for the Activation of Dynamic Recrystallization Caused by Thermal Cycles

Dynamic recrystallization caused by the hot deformation of stainless steel, such as 316 L, can be interpreted via the strain rate $\dot{\varepsilon }$ and temperature *T*, using the following parameter, known as the Zener–Hollomon parameter [39]:

(1) $$Z=\dot{\varepsilon }\times exp\left\{\frac{Q}{RT}\right\}$$

where *Q* = 460 kJ/mol [40] (for 316 L deformed around 1522 K) is the activation energy for triggering deformation and *R* = 8.314 J/mol⋅K is the universal gas constant.

Several reports show that when a critical strain is reached, dynamic recrystallization can be activated [41,42,43]. The relationship between the critical strain $\varepsilon_{c}$ for dynamic recrystallization and the Zener–Hollomon parameter can be obtained as follows [44]:

(2) $$\varepsilon_{c}=0.009\times Z^{0.084}$$

Dynamic recrystallization can be activated when the true accumulated strain reaches the critical strain. It can be seen from the above that the strain rate and temperature are the key factors to judge whether dynamic recrystallization occurs or not.

It is important to answer the question of how to determine whether the strain and temperature conditions caused by thermal cycles can trigger dynamic recrystallization. First of all, the strain rate $\dot{\varepsilon }$ is the derivative of the strain to time; the average strain rate in the thermal cycle time can be taken as the calculation parameter, and the thermal cycles time t of the 30 layers is 17 min. The strain condition is different from traditional dynamic recrystallization, as it contains the initial strain and the accumulated strain in DED. The initial strain is a stored strain caused by the solidification during the deposition of a layer, it can be expressed as dislocation density, as follows [45]:

(3) $$\varepsilon_{initial}=\frac{\sigma_{initial}}{E}=\frac{\alpha mGb\sqrt{\rho }}{E}$$

where α is the parameter of the interdislocation interaction, m is the orientation coefficient, G is the shear modulus of the matrix, and E is the elastic modulus of the material. For 316 L stainless steel, α=0.3, m=3, G=77.5 GPa, b=0.248 nm, E=200 GPa. The accumulated strain is a produced strain caused by heat conduction due to heating and cooling cycles in the adjacent N layers. It can be extracted from Figure 2b that the accumulated strain $\varepsilon_{accumulated}$ of 30/60/90 layers’ thermal cycles can be calculated to be about 2%, 4%, and 6%, respectively. Secondly, peak temperature T can be extracted from Figure 2a, the peak temperature T of 30/60/90 layers thermal cycles can be calculated to be about 1200 °C, 650 °C, and 600 °C, respectively.

In summary, the equation to determine whether the dynamic recrystallization is caused by thermal cycles can be rewritten as the following formula:

$$Z=\frac{\varepsilon_{initial}+\varepsilon_{accumulated}}{t}*exp\left\{\frac{Q}{RT}\right\}$$

(4) $$\varepsilon_{c}=0.009*Z^{0.084}$$

Dynamic recrystallization caused by thermal cycles can be activated when the true accumulated strain reached the critical strain. It is calculated to be about 0.17%, 8.8% and 9.2%, respectively, for the different numbers of layers. As a result, the true accumulated strain is greater than the critical strain under 30-layes thermal cycle conditions (<2%/1200 °C), but it is not reached under 60- and 90-layer thermal cycles (2–4%/650 °C and 4–6% /600 °C, respectively). This means that dynamic recrystallization occurs in the first 30 layers of the thermal cycle, but only recovery occur after that. The theoretical judgment is also consistent with the above phenomenon, which will be analyzed in detail below.

### 4.2. Solidification Mode of 316 L

**Figure 6:**
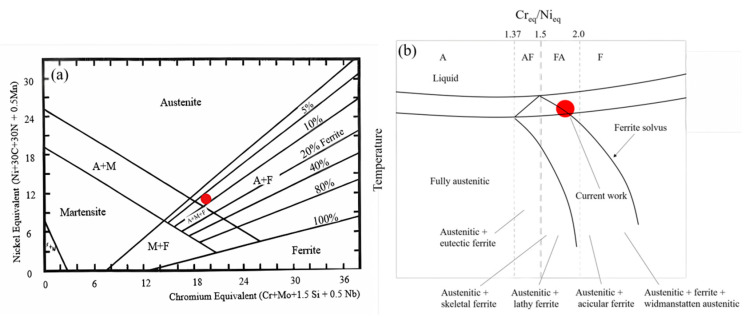
(**a**) The calculation method of Cr eq. and Ni eq. [46]. (**b**) Solidification mode and composition of 316 L stainless steel [26].

As mentioned in Section 3.3, δ-ferrite and γ-austenite are two common microstructural constituents in the solidification process of fast-cooled austenitic stainless steel, and their respective contents are determined by the solidification mode. This is based on Schaeffler and WRC-1992’s predictive graphs, as shown in Figure 6a, which only applies to austenitic stainless steels with a ferrite content between 0 to 100%. According to the conventional Schaeffler diagram [46], the calculation method of Cr eq. and Ni eq. is shown in Figure 6b, and the Cr- and Ni-equivalents can be calculated by Cr + Mo + 1.5Si + 0.5Nb and Ni + 30C + 30N + 0.5Mn, respectively. When the Cr eq./Ni eq. ratio is between 1.5 and 2.0, the solidification mode of stainless steel is in the FA region, The values calculated in this work are within the range of this area, as shown by the red dots in Figure 6b, while it enters the AF region when the Cr eq./Ni eq. ratio is reduced to between 1.37 and 1.5. It is worthwhile to consider the role of the cooling rate in different locations in the three groups mentioned above at an increasing distance from the substrate sample, although the cooling rates applied from the deposition temperature to room temperature in the DED 316 L are high enough in all regions compared to in traditionally fabricated steels. The FA region contains γ-austenite and δ-ferrite, with different shapes (i.e., skeletal ferrite and lathy ferrite), which reveals the diversity with the change in the Cr eq./Ni eq. ratio. Furthermore, the δ-ferrite will reduce with the decrease in the Cr eq./Ni eq. ratio, although it will remain in the FA region. Austenite and austenite + eutectic ferrite are included until the AF mode, which have less δ-ferrite.

### 4.3. Evolution of the Grain Size and Geometric Dislocation Density

Both stored energies caused by accumulated strain and temperature are needed to trigger dynamic recrystallization. Dynamic recrystallization occurs under all 30-layer thermal cycles conditions, as shown in Figure 3. The critical strain for dynamic recrystallization conditions is analyzed above in Section 4.1. In addition to the initial solidification microstructure, the grain size and GND are closely related to the strain and temperature caused by thermal cycles. Dynamic recrystallization nucleation results in the formation and migration of large-angle grain boundaries, which is divided into two steps. Firstly, nucleation occurs at the original large-angle grain boundary with accumulated strain as a driving force. At the same time, temperature fluctuation is used as an auxiliary factor to improve the ability of grain boundary migration. At the same time, dislocations migrate from the grain boundary by sliding, intertwining, and delivering each other, or are hindered by precipitates and the solid solution phase, and finally form sub-grains. Under the action of energy storage, the grain boundary gradually merges with the sub-grains brought by temperature recovery though the polygonization process, which, as the sub-grains coalesce and migrate, leads to the appearance of a high-angle boundary [47], forming a new grain nucleus, which has low-strain or strain-free region. The size of the initial grains, as well as the accumulated strain and temperature, also determine the difficulty of dynamic recrystallization. After the initial nucleation stage, only new grain growth occurs.

**Figure 7:**
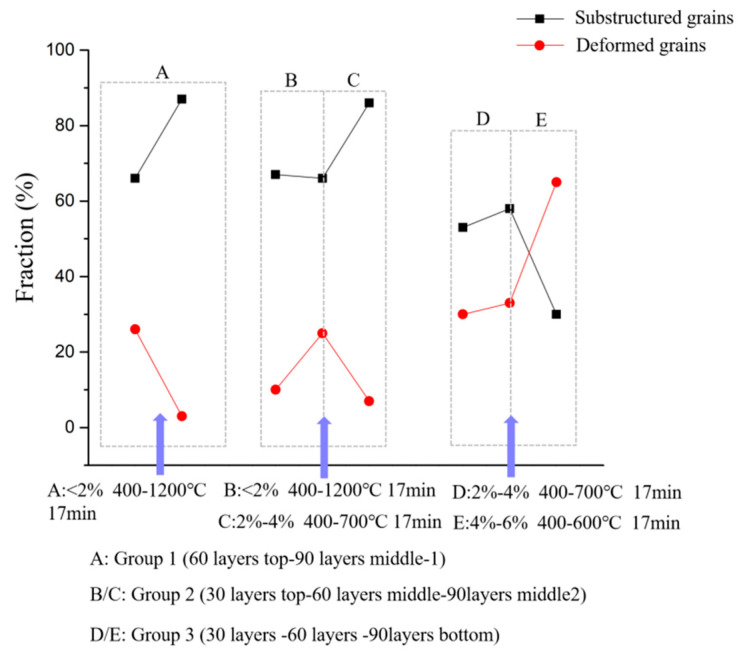
Sub-structured and deformed grains fraction diagram under various conditions including accumulated strain, temperature, and initial grain size caused by thermal cycles.

For group 1, the relatively large initial grain size (the top sample of 60 layers) reduces the driving force for dynamic recrystallization, and high temperature increases the activity of grain boundary migration, Under the combined effect of these two conditions, the critical strain of the dynamic recrystallization was 0.17%. As a result, the true accumulated strain (2%) is greater than critical strain under 30-layer thermal cycle conditions, as analyzed in Section 4.1. The sample undergoes a fully dynamic recrystallization. After grain nucleation and growth, coarse grains are formed (90 layers in the middle-1 sample), as shown in Figure 3e,i and as described in Section 3.2. Moreover, for alloy materials with low stacking fault energy, such as 316 L, a high temperature causes the dislocations to migrate from the large-angle grain boundary and to slip into the grain. The dislocations between different slip systems intertwine with each other, and the δ-ferrite formed by solidification has a strong pinning effect on the dislocations, resulting in dislocation rearrangement and the formation of new sub-grains [25]. These newly formed sub-grains contribute the increase in GND. In addition to the decrease in GND caused by grain coarsening due to the decline of grain boundaries, it is evidenced by the dotted box A in Figure 7 that the number of sub-structured grains increase while deformed grains decrease. This is the reason why the GND does not decrease much when the grains are obviously coarsened, as described in Section 3.3 and shown in Figure 4d,g.

For group 2, the initial grain size (top sample of 30 layers) is relatively small. As a result of the greater cooling rate, the ability of grains to resist deformation is relatively strong, which means that the initial strain $\varepsilon_{initial}$ increases before recrystallization. However, the calculated critical strain of dynamic recrystallization was also 0.17%. This means that the initial grain size is independent on the critical strain. Under the same accumulated strain (<2%) and temperature fluctuation (400–1200 °C) conditions with group 1, the driving force is sufficient to make the grains recrystallize, as analyzed in Section 4.1. However, the finer initial grain size results in a higher nucleation rate $\dot{N}$ and a higher number of nucleation sites at the original grain boundary, which leads to relatively fine recrystallized grains (the middle 60 layers), as shown in Figure 3c,f, described in Section 3.2, and analyzed in Section 4.2. Then, the peak temperature reduces to approximately 650 °C due to the distance from heat source for the 60-layer thermal cycle, which causes the critical strain $\varepsilon_{c}$ to increase to 8.8%, even though the accumulated strain gradually increases to 2–4%. As the actual strain does not reach the critical strain, the grain cannot be nucleated under the subsequent input of energy, and the increasing strain provides sufficient driving force to make the nucleation grain experience growth. As a result, coarse grains are formed (90 layers in the middle-2 section), as shown in Figure 3f,j. It is worth mentioning that in group 2, the GND does not decrease with the decline of grain boundary due to grain coarsening, but it slightly increases, as described in Section 3.3 and shown in Figure 4b,e,h. There are two reasons: one is the increase in the number of sub-grains as described above. As is evidenced by the dotted box B/C in Figure 7, the number of sub-structured grains increases, yet the deformed grains remain stable, which leads to the increase in GND. The other reason is that during solidification, δ-ferrite precipitated at the position of 30 layers in the top, 60 layers in the bottom, and 90 layers in the middle-2 section is higher than that of the sections with 60 layers at the top and 90 layers in the middle-1 section (Figure 5), which is caused by the slight difference of nucleation mode resulting from Cr eq./Ni eq. precipitation from different locations. As described in Section 3.4, δ-ferrite can impede the movement of dislocations activated by temperature and has a stronger pinning effect. This kind of pinning phenomenon can bring about more sub-grains, contributing to GND.

For group 3, the grains of the bottom sample of 30 layers are regarded as the initial grains, and these experience a 2–4% strain/400–650 °C temperature fluctuation for 17 min to create a 60-layer bottom sample, which then undergoes 4–6% strain/400–600 °C temperature fluctuation for 17 min to create a 90-layer bottom sample. The critical strains are 8.8% and 9.2%, respectively, which are greater than the actual strain. Dynamic recrystallization does not occur, and the grain size is almost stable (60- and 90-layer bottom). It can be seen that temperature is an important factor for recrystallization nucleation, and low temperatures would not promote recrystallization nucleation, even with an increasing strain energy input, as shown in Figure 3d,g,k and described in Section 3.2. Similarly, at the bottom of the three as-built samples, a large amount of δ-ferrite was formed during solidification (Figure 5), which provides enough pinning points for moving dislocations forming sub-grains due to appropriate strain and temperature, as described in Section 3.3 and shown in Figure 4c,f,i. It is also evident that the fraction of sub-structured grains increases, as in the dotted box D in Figure 7. What is more, under the relatively large strain (4–6%) condition, the formation of low LAGB comprised of regular dislocation arrays gradual transforms to HAGB. As a result, more LAGBs merge to form HAGB, which leads to a decrease in sub-structured grains and the promotion of deformed grains, as shown in the dotted box E in Figure 7, which also leads to the increase of GND. Therefore, GND increases after a 90-layer thermal cycle at the position of 90 layers from the bottom.

Other possible factors influencing dynamic recrystallization include solute segregation, second phase particles, and crystallographic texture. However, these characteristics scale inversely. What is more, the dynamic recrystallization hindrance brought about by solute segregation becomes less significant as samples are deformed up to higher strain levels [48].

**Figure 8:**
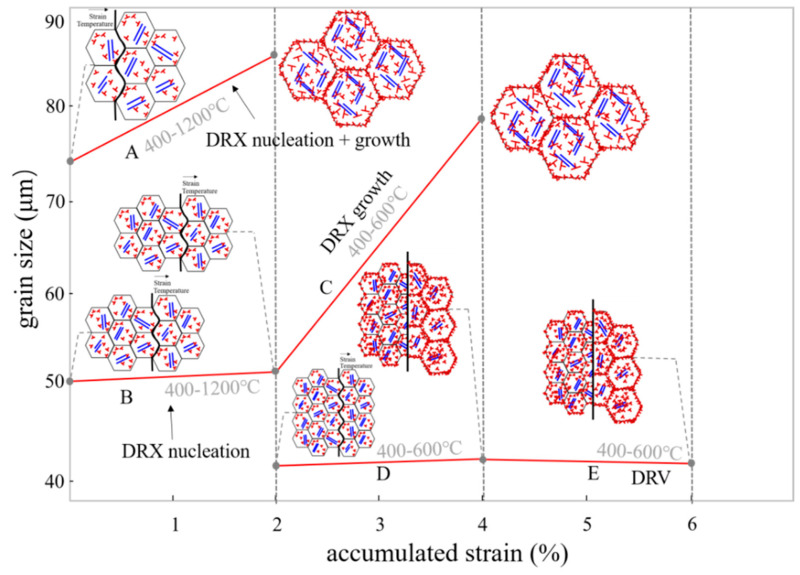
Transition map for the activation of dynamic recrystallization under various conditions including accumulated strain, temperature, and initial grain size caused by thermal cycles.

The accumulated strain in thermal cycles is actually caused by temperature fluctuations due to the expansion and contraction of the material. Thus, strain, temperature, and initial grain size are the three key factors affecting recrystallized grains. In the study of dynamic recrystallization induced by thermal cycles in this paper, large initial grain size combined with even low accumulated strain and high peak temperature causes fully dynamic recrystallization (nucleation + grain growth), which leads to coarse grains, as shown in line A of Figure 8. At the same strain and temperature, relatively small initial grains provide more nucleation sites and a greater nucleation rate for dynamic recrystallization, resulting in a stable grain size, as shown in line B of Figure 8. The decrease in temperature leads to the increase in the critical strain, which makes nucleation difficult. More strain input promotes grain growth after nucleation, resulting in coarse grains, as shown in line C of Figure 8. Similarly, line D and line E in Figure 8 show that dynamic recrystallization nucleation does not occur at low temperatures despite the increasing input strain energy. At this time, dynamic recovery occurs, and the grain size remains stable. Maintaining an appropriate temperature and strain can promote dislocation movement and form new sub-grains though thermal cycles, which leads to an increase in GND and in the strength of the as-deposited sample.

## 5. Conclusions

The present study investigated the effects of accumulated strain, temperature fluctuation, and initial grain size on the microstructural evolution during the thermal cycles process manufactured by DED, including the grain size and GND evolution process caused by the dynamic recrystallization, recovery, and precipitation of the solidified phase. The following conclusions can be drawn from the findings of the study:

(1) The initial grain size is inversely proportional to the cooling rate.
(2) The critical strain of dynamic recrystallization is determined by the accumulated strain and temperature during thermal cycling.
(3) Once the actual strain is greater than the critical strain, dynamic recrystallization occurs, and the grain size after dynamic recrystallization is related to the initial grain size, strain and temperature. The initial grain size affects the number of nucleation sites and the nucleation rate during the nucleation stage. Temperature affects the grain growth rate after nucleation by affecting the grain boundary mobility during the grain growth stage. Strain affects both the nucleation rate during the nucleation stage and the grain growth rate during the grain growth stage.
(4) Temperature, accumulated strain, and the amount of δ-ferrite affect the formation of sub-grains during dynamic recrystallization caused by thermal cycles. High temperatures can promote dislocation movement, and precipitated δ-ferrite can pin moving dislocations. The trapped dislocations form new sub-grains though thermal cycles, which leads to the increase in GND.

## Figures and Tables

**Table 1:** Chemical composition of 316 L stainless steel.

Element	Cr	Ni	Mo	C	Mn	N	Si	Fe
Percentage (%)	16.28	12.05	2.1	0.021	1.54	0.005	0.68	Bal

## Data Availability

The original contributions presented in the study are included in the article, further inquiries can be directed to the corresponding author.
